# Quantifying Biochemical Alterations in Brown and Subcutaneous White Adipose Tissues of Mice Using Fourier Transform Infrared Widefield Imaging

**DOI:** 10.3389/fendo.2017.00121

**Published:** 2017-05-31

**Authors:** Ebrahim Aboualizadeh, Owen T. Carmichael, Ping He, Diana C. Albarado, Christopher D. Morrison, Carol J. Hirschmugl

**Affiliations:** ^1^Department of Physics, University of Wisconsin-Milwaukee, Milwaukee, WI, United States; ^2^Pennington Biomedical Research Center, Baton Rouge, LA, United States

**Keywords:** Fourier transform infrared imaging, spectroscopy, obesity, brown adipose tissue, subcutaneous white adipose tissue, adipose thermogenic markers

## Abstract

Stimulating increased thermogenic activity in adipose tissue is an important biological target for obesity treatment, and label-free imaging techniques with the potential to quantify stimulation-associated biochemical changes to the adipose tissue are highly sought after. In this study, we used spatially resolved Fourier transform infrared (FTIR) imaging to quantify biochemical changes caused by cold exposure in the brown and subcutaneous white adipose tissues (BAT and s-WAT) of 6 week-old C57BL6 mice exposed to 30°C (*N* = 5), 24°C (*N* = 5), and 10°C (*N* = 5) conditions for 10 days. Fat exposed to colder temperatures demonstrated greater thermogenic activity as indicated by increased messenger RNA expression levels of a panel of thermogenic marker genes including uncoupling protein 1 (UCP-1) and Dio2. Protein to lipid ratio, calculated from the ratio of the integrated area from 1,600 to 1,700 cm^−1^ (amide I) to the integrated area from 2,830 to 2,980 cm^−1^ (saturated lipids), was elevated in 10°C BAT and s-WAT compared to 24°C (*p* = 0.004 and *p* < 0.0001) and 30°C (*p* = 0.0033 and *p* < 0.0001). Greater protein to lipid ratio was associated with greater UCP-1 expression level in the BAT (*p* = 0.021) and s-WAT (*p* = 0.032) and greater Dio2 expression in s-WAT (*p* = 0.033). The degree of unsaturation, calculated from the ratio of the integrated area from 2,992 to 3,020 cm^−1^ (unsaturated lipids) to the integrated area from 2,830 to 2,980 cm^−1^ (saturated lipids), showed stepwise decreases going from colder-exposed to warmer-exposed BAT. Complementary ^1^H NMR measurements confirmed the findings from this ratio in BAT. Principal component analysis applied to FTIR spectra revealed pronounced differences in overall spectral characteristics between 30, 24, and 10°C BAT and s-WAT. Spatially resolved FTIR imaging is a promising technique to quantify cold-induced biochemical changes in BAT and s-WAT in a label-free manner.

## Introduction

Brown adipose tissue (BAT), defined by the presence of uncoupling protein 1 (UCP-1), has the potential for a higher metabolic rate than the far more prevalent white adipose tissue (WAT) in mammals, due to its propensity for thermogenesis. For this reason, increasing metabolic activity within the BAT, and encouraging BAT-like metabolic activity within the WAT, a prime target for increasing adipose tissue energy expenditure and thus promoting weight loss and healthy weight maintenance in humans (1). A variety of pharmacological agents and prolonged cold exposure increase the metabolic rate of adipose tissue, either by increasing classical non-shivering thermogenic activity within the BAT or by inducing BAT-like thermogenic activity within certain adipocytes (“beige” or “brite” cells) within the WAT (2–4). Besides displaying increased energy expenditure, mice with increased BAT or WAT metabolic activity also show increased insulin sensitivity (5), suggesting reduced risk of type 2 diabetes.

Numerous tomographic imaging techniques have been used to determine the metabolic activity of adipose tissue. Positron emission tomography (PET) with fluorodeoxyglucose (FDG) (6–9) is the primary method to assess BAT in humans. Although FDG PET is widely available, ionizing radiation exposure, cost, procedure duration, and poor reproducibility of images are its key limitations (10–12). Computed tomography scanning (13) provides a static snapshot of body composition, including differences between adipose tissue and other tissues, but as a macroscopic technique it has limited ability to assess precise biochemical properties and as a static technique it is unable to capture dynamic changes to adipose tissue metabolism. Hyperpolarized ^13^C MRI (14) and hyperpolarized xenon MRI (15) are promising for providing dynamic biochemical measurements in adipose tissue, but these techniques require costly and complex hyperpolarization procedures. BOLD fMRI (16) provides dynamic and non-invasive measurements of blood oxygenation as a proxy for adipose tissue metabolic activity, but it is unable to dissociate changes in blood flow from changes in adipose tissue metabolism.

*In vivo* techniques outside of tomographic imaging have also been used in this subject. Indirect calorimetry (17) is a non-invasive method that permits measurement of whole-body energy expenditure, but it does not permit quantification of energy expenditure within specific tissues such as BAT and subcutaneous WAT (s-WAT). Infrared (IR) thermography (18), a thermal imaging technique, has been developed for detecting BAT, but the technique is limited by confounding effects of skin, vasculature, and other surface tissues covering the BAT; in addition as a photography technique it lacks the ability to provide measurements deep below the body surface.

*Ex vivo* microscopy is also widely used to assess adipose tissues. A wide variety of immunohistochemical stains including hematoxylin and eosin, horseradish peroxidase substrates, and antigen labeling with a fluorochrome-conjugated antibody have been adapted for analyzing paraffin-embedded and formalin-fixed adipose tissue sections (19, 20). However, fixation and staining can distort the architecture and the metabolic state of the adipose tissue, in some cases leading to a biased assessment of the native biochemistry of the tissue (21). Due to the limitations of these existing techniques and the high scientific relevance of adipose tissue metabolism, there is an ongoing need to develop alternative imaging methods to quantify adipose tissue structural and dynamic characteristics.

Fourier transform infrared (FTIR) spectroscopic imaging (22) is a label-free and non-destructive technique that quantifies the distribution of biologically relevant components in samples, concurrently revealing biochemical composition and morphology. FTIR imaging permits detecting the inherent vibrational mid-IR spectra of the biochemical constituents of cells and characterization of localized biochemical changes. In this article, for the first time, we demonstrate that FTIR imaging is a promising approach to quantifying biomolecular changes attributed to cold acclimation in BAT and s-WAT excised from mice. The potential of FTIR imaging to measure intact tissues (23, 24) and living cells (25–28) without applying exogenous labels has been thoroughly studied. FTIR imaging has been previously used by obesity researchers to quantify the fatty acid composition of human abdominal fat (29), as well as obesity-induced alterations in liver and muscle tissues in BXD recombinant inbred mice (30), determination of the lipid profile of liver tissue (31), and obesity-induced alterations in subcutaneous and visceral adipose tissues (32). To our knowledge, none of these prior works demonstrate that FTIR imaging is capable of detecting biochemical changes to the BAT and s-WAT caused by cold exposure.

In this study, we used FTIR imaging to assess BAT and s-WAT derived from mice exposed to 30°C (thermoneutral condition), 24°C (room temperature maintained), and 10°C (cold exposed). The ability of cold exposure to stimulate adipose tissue metabolism was evaluated by quantitative measurement of messenger RNA (mRNA) expression levels of a panel of marker genes. We performed FTIR microspectroscopy to identify regions of interest within each tissue sample that had varying FTIR signal properties, in order to characterize the full breadth of possible FTIR signals within the tissue. We then performed FTIR widefield imaging within each of these diverse regions to characterize the chemical distribution of macromolecules within them. Principal component analysis (PCA) was then used to delineate spectral features that discriminated the full range of diverse regions between temperature groups and between fat depots. Complementary ^1^H NMR measurements were performed to validate group differences identified by FTIR wide field imaging.

## Materials and Methods

### Animals

Male C57BL6 mice (Jackson Laboratories) were purchased at 6 weeks of age and were given a standard low (10%) fat diet (WD-C or HFL-C; 98052602, D12450B) and were housed at room temperature (24°C) in standard caging (four per cage) with a 12:12 light:dark cycle and food and water provided *ad libitum*. In this study, there were three groups of five animals each. Each group was exposed to one of the three temperatures studied. All diets were purchased from Research Diets (New Brunswick, NJ). At week 8, all animals were housed (four per cage) at thermoneutrality (30°C) to minimize thermogenic activity for 1 week. At week 10, each animal was moved from four per cage to single housing to acclimate for 1 week in a single-housed environment. Then, each animal was randomized to one of three treatment groups: (1) thermoneutral (30°C), (2) room-temperature maintained (24°C), and (3) cold exposed (10°C). Each animal was transitioned from thermoneutrality to single housing at the new ambient temperature for 7 days prior to sacrifice to induce varying degrees of fat activation in response to cold. Adipose tissue extraction and metabolic measurement in the mice follows a protocol that was described previously (33). Briefly, mice were euthanized by exposure to lethal dose of CO_2_. Adipose tissues from BAT and s-WAT compartments were collected by surgical excision and the tissue was divided into two portions. One portion of the dissected BAT and s-WAT was immediately frozen in isopentane cooled to almost freezing in liquid nitrogen and later stored at −80°C for FTIR, and nuclear magnetic resonance measurements. Freezing was performed expediently to preserve tissue integrity and metabolic state. The other portion was preserved for assessing gene expression for key thermogenic markers at Pennington Biomedical Research Center. Tissue used for RNA extraction was immediately snap-frozen in liquid nitrogen to ensure optimal RNA integrity. Samples went through DNAase treatment during RNA isolation. Also, “no reverse transcriptase (RT)” controls are included in RT and qPCR to prevent gDNA contamination of each assay. All of those controls had no signal, like the no template controls, in the qPCR results. All procedures were in accordance with the National Institutes of Health Guide and animal experiments were approved by the Institutional Animal Care and Use Committee of Pennington Biomedical Research Center.

### Measurement of Adipose Thermogenic Markers

To confirm the effectiveness of cold exposure to activate BAT and remodel s-WAT, gene expression for key thermogenic markers (UCP-1, DIO2, CIDEA) (2) was assessed via real-time PCR within the PBRC Cell Biology and Bioimaging Core on an ABI 7900HT sequence detector. RNA extraction and real-time PCR was conducted as described previously (34). Total RNA was extracted from liver, s-WAT, and BAT using TRIzol reagent following the manufacturer’s protocol (15596018, Invitrogen) and Qiagen Lipid Tissue RNeasy mini kit (Qiagen Cat# 74804). RNA quality and quantity was determined by spectrophotometry using a NanoDrop (Thermo Scientific). cDNA synthesis was performed with a highly efficient RNase H+ MMLV RT (BioRad Cat# 1708890 iScript cDNA Synthesis kit). mRNA was quantified on the ABI 7900 Real Time qPCR platform using the SYBR green methodology with standard curve analysis in optical 384-well plates (Applied Biosystems) in triplicates using the SYBR Green PCR Master Mix (ABI Cat# 4309155) protocol. Primer pairs were designed using IDT Real Time PCR primer design, UCSC Genome Browser *in silico* PCR tool, and NCBI Primer-BLAST with amplicon and primers spanning exon–intron boundaries. Target gene expression was normalized with cylcophilin (ppia) as the endogenous control, which has been shown to have consistently stable and unchanged gene expression in the tissue measured under the experimental parameters used for the data presented. Primers sequences are as follows:

Cyclophilin (ppia) F: 5′-CTTCGAGCTGTTTGCAGACAAAGT-3′; R: 5′ AGATGCCAGGACCTGTATGCT-3′
Ucp-1 F: 5′-CACCTTCCCGCTGGACACT-3′; R: 5′-CCCTAGGACACCTTTATACCTAATGG-3′ Cidea F: 5′-ATCACAACTGGCCTGGTTACG-3′; R: 5′-TACTACCCGGTGTCCATTTCT-3′ Dio2 F: 5′-CAGTGTGGTGCACGTCTCCAATC-3′ R: 5′-TGAACCAAAGTTGACCACCAG-3′.

### Sample Preparation for FTIR Measurements

Brown adipose tissue and s-WAT stored at −80°C, were transported to the biotechnology facility (University of Wisconsin-Milwaukee) for sectioning using a cryostat machine (Leica Model CM 3050S). All tissue was cut at a temperature between −15 and −20°C inside the cryostat, and the blade was pre-chilled at least 30 min before sectioning. Adipose tissues were embedded in an optimal cutting medium (OCT) compound (Sakura Finetek Inc., USA) and the cryomold was left in the cryostat machine (at −20°C) for 1 min prior to sectioning. Only small amount of OCT was applied (underneath the sample) to prevent tissue contamination. The position of the tissue was adjusted to be near the blade in the desired orientation and an appropriate thickness was chosen (5–8 µm for FTIR measurements). At least eight sections from each tissue sample were collected, and each section was mounted on a mid-IR BaF_2_ window for transmission measurements. Prior to FTIR measurements, the sections were thawed (desiccated) in a dark box and warmed to room temperature in low humidity. In desiccation drying, we usually leave the tissue for at least 2 h to make sure that the tissue is fully dried, because any left over water on the tissue damages the tissue integrity. Because the sections were light sensitive, exposure to visible light outside of the FTIR instrument was minimized.

#### FTIR Microspectroscopy

Fourier Transform Infrared spectra of BAT and s-WAT (an area of approximately 1.5 × 1.5 mm^2^) were recorded using a Bruker vertex 70 IR spectrometer bench coupled to an IR microscope equipped with a single element Mercury Cadmium Telluride detector. FTIR spectra were collected in transmission mode using a 15× Cassegrain microscope objective (numerical aperture 0.4) and a 15× Schwartzchild condenser (numerical aperture 0.4) for single point measurements. The spectrometer is attached to a microscope and a computerized stage equipped with a CCD camera that permits collecting bright-field images of the sampling area. Each FTIR spectrum was collected over a tile of the section covering 140 × 140 μm^2^. Moving the motorized stage allowed us to collect data from multiple tiles covering the entire tissue section (roughly 100 tiles per section). Each FTIR spectrum represented an average of 128 coadded scans in the wavenumber region between 4,000 and 650 at 4 cm^−1^ spectral resolution. Background scans were collected from a no sample region and ratioed against the sample spectrum.

### FTIR Widefield Imaging

Individual tiles within each section were selected for FTIR widefield imaging using a data analytic method that identified a set of tiles displaying the greatest diversity in the FTIR microspectroscopy signal. The tiles with the most varying signal within each tissue section were identified by PCA, which decomposes the data in the order of importance of variance (see Principal Component Analysis for Identification of Regions of Interest Within BAT and s-WAT section). The focal plane array (FPA)-FTIR images of adipose tissue sections were recorded with the use of a Bruker vertex 70 IR spectrometer coupled with a Bruker Hyperion 3000 IR microscope. Images were acquired by means of a multielement (64 × 64 pixels) FPA detector. The FPA size for the measurements was set to 64 × 64 pixels; therefore, 4,096 individual spectra in the mid-IR wavelength range 3,800–900 cm^−1^ (every pixel contains an IR spectrum) were collected per single tile measurement. The measurements were performed using a 15× Cassegrain microscope objective and a 15× condenser aperture. This experimental geometry allows us for high magnification imaging capabilities with a 2.2 × 2.2 µm^2^ pixel size and a 140 × 140 µm^2^ FOV. We used 512 scans coadded at a spectral resolution of 4 cm^−1^ with a zero filling factor 2 for background and for sample acquisitions, respectively. To cover a larger area of the tissue, four adjacent tiles were measured by mapping across the section of the adipose tissue. Each image covered an area of 280 × 280 μm^2^ (each image = 2 × 2 tiles; each tile = 64 × 64 pixels; each pixel = 2.2 µm).

### ^1^H NMR Spectroscopy Sample Preparation

Brown adipose tissue and s-WAT samples stored at −80°C were extracted using a mixture of MTBE (methyl tert-burtyl ether), methanol, and water (35, 36). A 50 mg sample of frozen tissue was homogenized by adding 1 mL of methanol and grinding with a hand tissue grinder. 4 mL of MTBE was added to the homogenized tissue and the samples rested on ice for an hour. Phase separation was accomplished by adding an additional 1 mL of water and centrifuging at 4°C for 15 min. Then, the organic phase (upper layer) was collected and combined with a second extraction, which was performed on the leftover pellet using the same method. The final organic phase was dried over a speed vacuum and prepared for NMR analysis. CDCl3/CD3OD/D2O (60:30:4.5, v/v/v) was used as a cosolvent in order to suppress the generation of large lipid vesicles, which would lead to severe proton line broadening.

### ^1^H NMR Data Acquisition and Processing

^1^H NMR measurements were performed using 500 MHz spectrometer (Minispec, Brucker Optics, Billerica, MA, USA). A pre-saturation pulse was used to saturate the water residual signal. The water frequency was selected as the carrier frequency and a lower power long pulse was applied to saturate water spins before excitation. A 90° hard pulse was applied to all proton spins, followed by detection. To enhance the accuracy of quantitative analysis, a 30-s recycle delay was used. This allowed all spins to relax before the next excitation. Collected data were processed using the MestReNova software package. The proton peak of the solvent CDCl3 was used as an internal reference (7.28 ppm). No line broadening was applied and all spectra used the same zero- and first-order phase correction.

### Data Analysis

#### PCA for Identification of Regions of Interest within BAT and s-WAT

Principal component analysis (R version 3.2.1) was performed on 100 spectra collected by FTIR microspectroscopy per tissue section, where each spectrum represented an area of 140 × 140 μm^2^ in the tissue. FTIR spectra were pre-processed (Matlab 2016) prior to PCA as follows. The CO_2_ peak at 2,350 cm^−1^ was flattened between 2,500 and 2,200 cm^−1^. The baseline was corrected by fitting a linear regression line to spectral points in the 2,692–1,920 cm^−1^ range, and subtracting that line from the spectrum. Then, the signal to noise ratio of every spectrum was systematically assessed by defining the noise content as the standard deviation in the 2,000–1,900 cm^−1^ spectral region, and the signal as the maximum of the signal between 3,100 and 2,750 cm^−1^. PCA is an unsupervised method that provides a set of principal components representing modes of maximum variance within the data set, as well as a set of scores describing where the original data points lie in the space defined by the principal components. Plotting these scores enables visualization of the underlying similarities and differences among data points in a low-dimensional space. PCA loadings plot highlights the major variables (wavenumbers) that contribute the most to the total variance in the data, described by that principal component. When major discriminating wavenumbers were identified from loadings, the absorption strengths of the identified wavenumbers were evaluated within each tile. The tiles with higher absorption values at determined wavenumbers were found and selected for FTIR widefield imaging.

#### PCA for Assessing Intergroup Differences in FTIR Images of BAT and s-WAT

Principal component analysis was applied to classify the spectra from different temperature tissues. From each tissue section, we derived spectra from each pixel of the FTIR images (600 spectra) and pre-processed (see Principal Component Analysis for Identification of Regions of Interest Within BAT and s-WAT section) for importing into PCA algorithm. Each spectrum represented an area of 2.2 × 2.2 μm^2^ in the tissue (i.e., every pixel within an image contains an FTIR spectrum). The comparison was made between 10, 24, and 30°C BAT and s-WAT to ascertain the biochemical differences between temperature groups. Segregation of spectra in the PC space and the wavenumbers associated with the classification between spectra were reported.

#### Statistics

We used one-way ANOVA models with *post hoc F* test to assess the differences in the molecular factors. Protein to lipid ratio is a critical parameter that provides qualitative information on the relative protein content in the adipose tissue (37). This ratio was obtained as a ratio of the integrated area of the amide I band of proteins (1,650 cm^−1^) to the integrated area of the saturated lipid content in C-H region (2,830–2,980 cm^−1^). The degree of unsaturation was assessed by calculating the ratio of the area of the olefinic band (2,992–3,020 cm^−1^) to the saturated lipid content in C-H region (2,830–2,980 cm^−1^). This ratio indicates the relative content of unsaturated lipids and double bonds in the lipid structure of the tissue (38). The spectral regions 1,750–1,500 cm^−1^ (for protein band) and 3,050–2,750 cm^−1^ (for saturated lipid bands) were used. *p* values less than 0.05 were accepted as statistically significant. In addition, linear regression models were used to determine linear relationships between expression levels of UCP-1, and the FTIR-based protein to lipid ratio. The least square means and standard errors were calculated for each treatment (10, 24, and 30°C). The least square mean values, associated *p*-values and *R*^2^ values from the comparisons, were reported for BAT and s-WAT. The statistical analysis and regression models were performed in SAS 9.4.

## Results

### Adipose Thermogenic Markers in Cold-Exposed Mice

**Figure 1:**
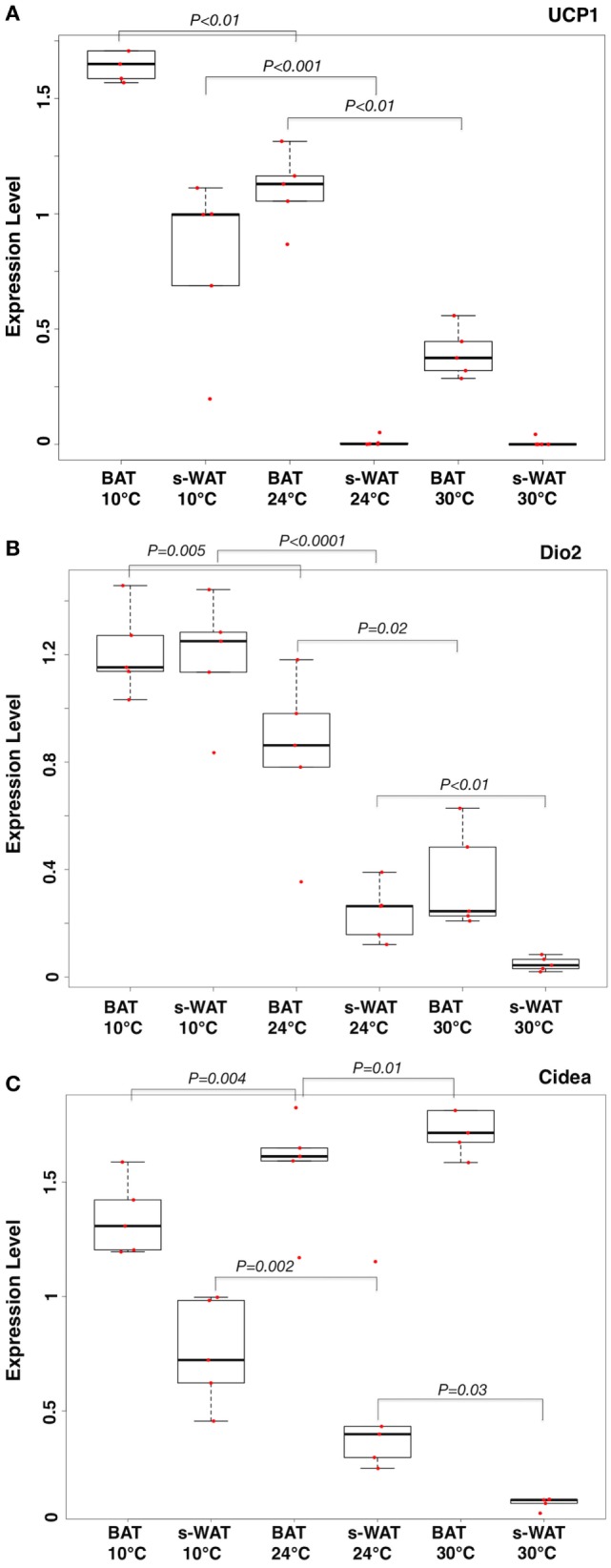
Boxplots showing the relative expression levels of uncoupling protein 1 (UCP-1) **(A)**, Dio2 **(B)**, and Cidea **(C)** in brown adipose tissue (BAT) and subcutaneous white adipose tissue (s-WAT) for individual data points measured from each animal at 10, 24, and 30°C is shown. Statistical analysis of adipose thermogenic markers between temperature groups was assessed by ANOVA followed by *post hoc F*-test and *p*-values were reported. Stepwise increase in UCP-1 level going from 30 to 24 to 10°C BAT (*p* < 0.01) was reported. UCP-1 level in 10°C s-WAT was greater than 24 and 30°C s-WAT (*p* < 0.001). *p* < 0.05 were accepted as the level of significance. *N* = 5; *N* represents the number of animals per adipose tissue/temperature.

One-way ANOVA models suggested that expression levels of three key mRNA indicators of fat activation (UCP-1, Cidea, and Dio2) within WAT, as well as levels of UCP-1 in BAT, increase in a stepwise fashion going from 30 to 24 to 10°C groups (Figure 1). Specifically, expression levels of all mRNA were significantly greater in the 10°C group compared to the 30°C group (all *p* < 0.05). Expression levels of all markers except Cidea in s-WAT were significantly greater in the 10°C group compared to the 24°C group (all *p* < 0.05), and levels of UCP-1 in BAT and Cidea in s-WAT are significantly greater in the 24°C group compared to the 30°C group (Figure 1A). Figures 1A–C show the boxplots of the relative gene expression levels (UCP-1, Dio2, and Cidea) calculated for individual data points at 30, 24, and 10°C.

### Identification of Regions of Interest Using FTIR Microspectroscopy

**Figure 2:**
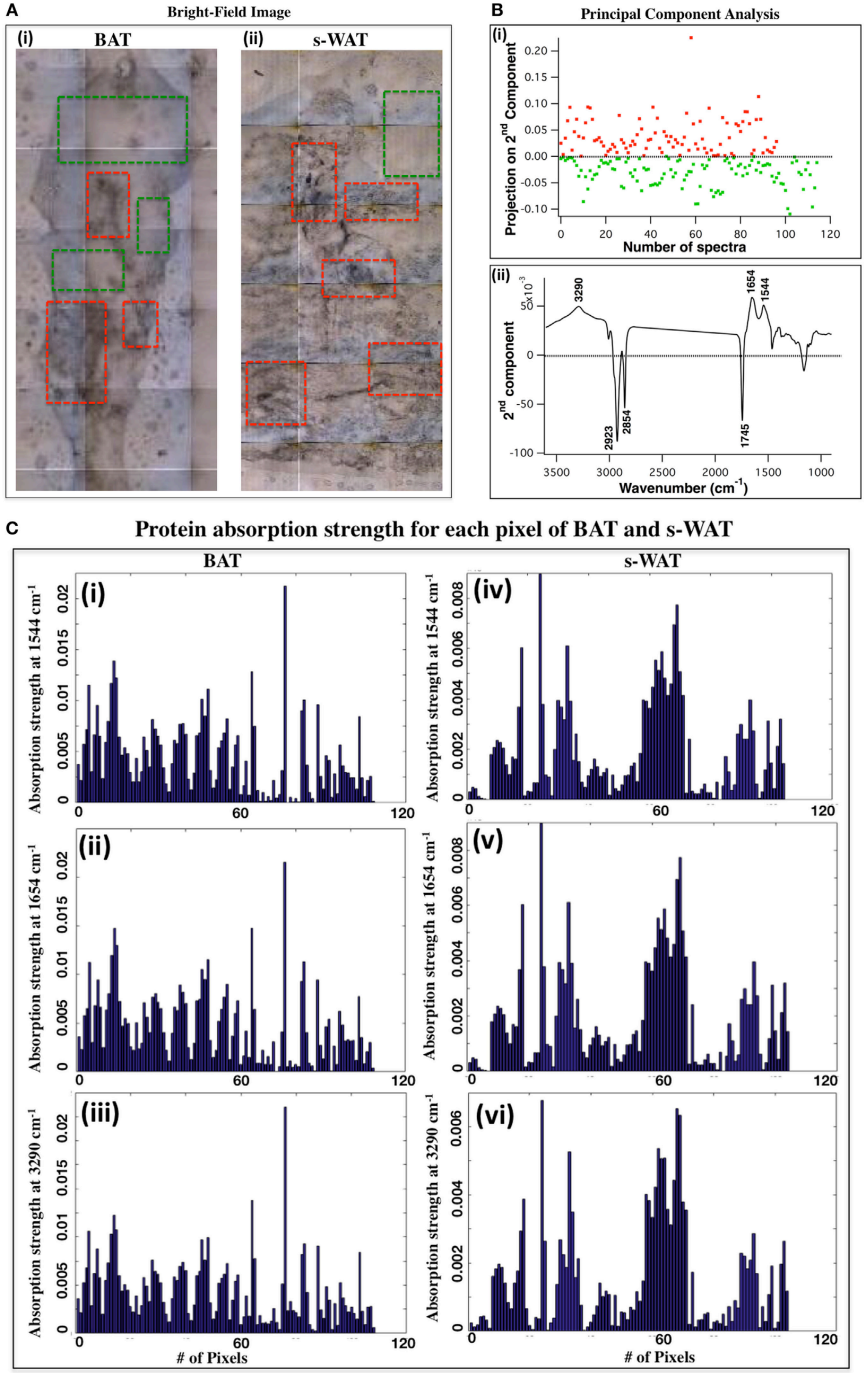
selection of regions of interest within adipose tissue using Fourier transform infrared (FTIR) microspectroscopy and principal component analysis (PCA). **(A)** Bright-field image of a cold-exposed brown adipose tissue (BAT) [**(A)**, i] and subcutaneous white adipose tissue (s-WAT) [**(A)**, ii] are shown. Data are representative. *N* = 5; *N* represents the number of animals per adipose tissue/temperature. Red boxes show the representative regions with different morphological appearances within tissue section and green boxes show more homogeneous regions within tissue. **(B)** Projection of spectra on the second component (scores) from PCA [**(B)**, i] and associated loading plot [**(B)**, ii] with the greatest varying wavenumbers are shown. Nearly 100 spectra from each tissue section (one representative section is shown in this figure) were pre-processed and loaded into PCA. Positive bands in PC-2 loading plot were attributed to proteins (1,544, 1,654, and 3,290 cm^−1^), and the negative bands (1,745, 2,854, and 2,923 cm^−1^) were attributed to lipids [**(B)**, ii]. Along PC2, positive scores are shown in red and negative scores are shown in green [**(B)**, i]. Red (green) scores were representative of the regions similar to the red (green) boxes that were superimposed on the bright-field images **(A)**. **(C)** Absorption strength of protein bands for the spectra that were generated from examination of the representative BAT [(i), (ii), and (iii)] and s-WAT [(iv), (v), and (vi)] (area: 1.5 mm × 1.5 mm) using FTIR microspectroscopy are shown. The tiles with the most varying FTIR signals were selected for subsequent FTIR imaging.

Figure 2A shows the bright-field images of 10°C BAT (i) and s-WAT (ii) and some tissue regions with different morphological appearances are highlighted (red boxes). Figure 2B shows PCA results from the spectra that were derived from FTIR microspectroscopy experiments (see FTIR Microspectroscopy and PCA for Identification of Regions of Interest within BAT and s-WAT for details). Projected scores on the second components [Figure 2B—Panel (i)] and associated loading plot [Figure 2B—Panel (ii)] are shown. The plot of scores facilitates the identification of spectra and inspection of loading plot allows us to determine which peaks contribute the most in distinguishing the spectra (Figure 2B). The loading plot from PCA [Figure 2B—Panel (ii)] shows three positive bands at 3,290, 1,654, and 1,544 cm^−1^ (protein bands) and three negative bands at 2,923, 2,854, and 1,745 cm^−1^ (lipid bands). The major wavenumbers responsible for the classification between the spectra were attributable to proteins. Since, these spectra were generated from the entire tissue (representative data from one section of BAT and s-WAT), protein bands were representative of the maximum spectral variations in the tissue section, along the second component. The positive scores (projection of spectra) along the second component [Figure 2B—Panel (i)] were identified and the tiles within each tissue section, associated with these positive scores were determined. Scores in red color were attributed to the regions similar to the superimposed red boxes in Figure 2A and the green scores were attributed to the regions similar to the green boxes in Figure 2A. Figure 2C displays the absorption strength of the three protein bands (3,290, 1,654, and 1,544 cm^−1^) for each tile (140 × 140 μm^2^) within the entire BAT [Figure 2C—Panels (i), (ii), and (iii)] and s-WAT [Figure 2C—Panels (iv), (v), and (vi)] sections.

### FTIR Widefield Imaging within Identified Regions of Interest

**Figure 3:**
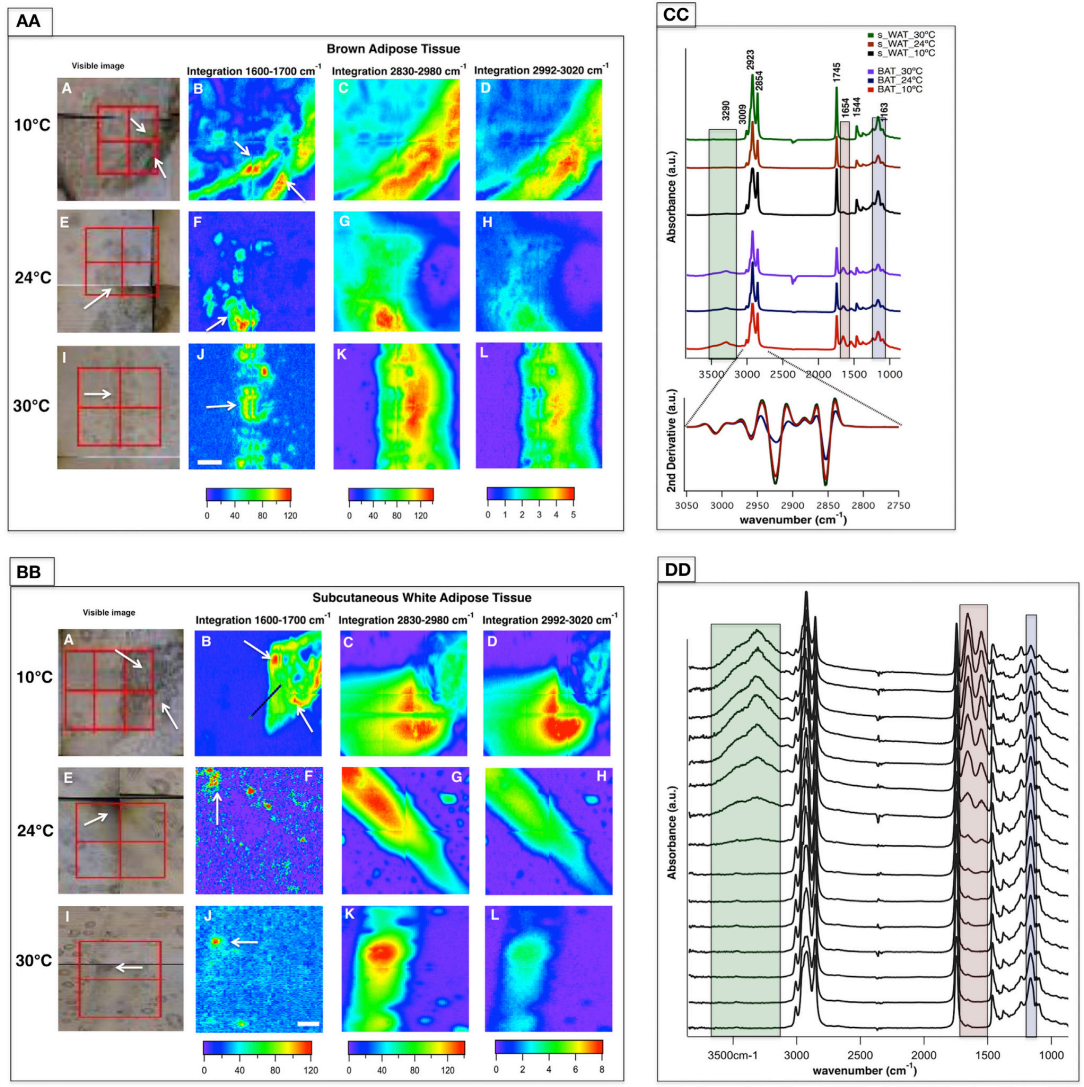
Fourier transform infrared (FTIR) images derived from brown adipose tissue (BAT) **(AA)** and subcutaneous white adipose tissue (s-WAT) **(BB)** at 10, 24, and 30°C are shown. Data are representative of one tissue section. *N* = 5; *N* represents the number of animals per adipose tissue/temperature. Panels A, E, and I are bright-field images of adipose tissues. Each tissue covers the area of 280 × 280 µm^2^ (2 × 2 tiles; each tile = 64 × 64 pixels; each pixel = 2.2 × 2.2 µm^2^). FTIR images from integrated area under 1,600–1,700 cm^−1^ associated with proteins (Panels B, F, and J), 2,830–2,980 cm^−1^ associated with saturated lipids (Panels C, G, and K), and 2,992–3,020 cm^−1^ associated with unsaturated fatty acids (Panels D, H, and L) are shown. Colors indicate component gradients from low (blue) to high (red) and the values are shown below each panel. The scale bar is 40 µm. **(CC)** Average of nearly 1,000 pixel spectra (3,600–900 cm^−1^) and a corresponding second derivative spectra (3,050–2,750 cm^−1^) for BAT and s-WAT at 10, 24, and 30°C are shown. **(DD)** Stack of spectra from the linescan drawn in **(BB)**/Panel B is shown. The starting spectrum in the stack (from bottom) is the spectrum derived from the green dot shown in [**(BB)**/Panel B]. The protein regions of the spectra are highlighted with pink, and green rectangles in Panels CC and DD to show the differences in protein absorbance.

Representative data from one section of BAT and s-WAT at each temperature group (10, 24, and 30°C) are shown. Bright-field images of BAT/s-WAT sections at 10°C (Figures 3AA,BB—Panel A), 24°C (Figures 3AA,BB—Panel E), and 30°C (Figures 3AA,BB—Panel I) are shown. Spectral maps were generated by integrating over different spectral regions including 1,600–1,700 cm^−1^ [proteins-amide I (Panels B, F, and J)], 2,830–2,980 cm^−1^ [lipids—CH_2_ and CH_3_ stretching (Panels C, G, and K)], and 2,992–3,020 cm^−1^ [olefinic—unsaturated fatty acids (Panels D, H, and L)]. Distribution maps were derived from four adjacent tiles (2 × 2 tiles), covering the area of about 280 × 280 μm^2^ within the section of a tissue. Chemical images show the relative concentrations of each functional group on a rainbow color scale [red (blue)/highest (lowest) intensity] to correspond to the absorption intensity and therefore concentration. The blue/purple background denotes the lack of biological material, while the green to red distributions shows elevated levels. The segments of the adipose tissues marked with white arrows in the visible images (Figures 3AA,BB—Panels A, E, and I) were detected by FTIR imaging (Figures 3AA,BB—Panels B, F, and J).

Figure 3CC demonstrates IR average spectra from BAT and s-WAT (30, 24, and 10°C) that highlight the IR bands attributable to carbohydrates, proteins, and lipids. Lipids dominate the mid-IR spectral region 3,050–2,800 cm^−1^. The band at 3,009 cm^−1^ attributed to olefin and unsaturated fatty acid (39) was observed in both BAT and s-WAT. Similarly, the four lipid bands at 2,854 (ν_s_ CH_2_), 2,873 (ν_s_ CH_3_), 2,923 (ν_as_ CH_2_), and 2,960 cm^−1^ (ν_as_ CH_3_), respectively (40), were observed in the spectra from both BAT and s-WAT. Clear differences in spectrum content, in particular in the heights of characteristic protein peaks in the spectral regions 3,600–3,100 cm^−1^ attributed to N-H and O-H stretching, amide I (1,700–1,600 cm^−1^) and amide II (1,570–1,500 cm^−1^) peaks, between BAT and s-WAT were observed. The highlighted green, pink, and blue boxes represent the variations in the absorption strength of the bands between the tissues (Figure 3CC). Absorption bands at 1,163 cm^−1^ attributed to C-O-C stretching of the ester functionalities (40) and 1,745 cm^−1^ assigned to C=O stretching of the carbonyl group are also seen in the spectra from BAT and s-WAT. Figure 3DD shows the stack of spectra along the linescan drawn in the Figure 3BB/Panel B. The first eight spectra in Figure 3DD show lack of protein absorptions (highlighted rectangles); however, the rest of the spectra show elevated absorption of the protein bands at 3,290, 1,654, and 1,544 cm^−1^. The stack of spectra clearly shows the differences in the protein profile in different segments of the tissue.

**Figure 4:**
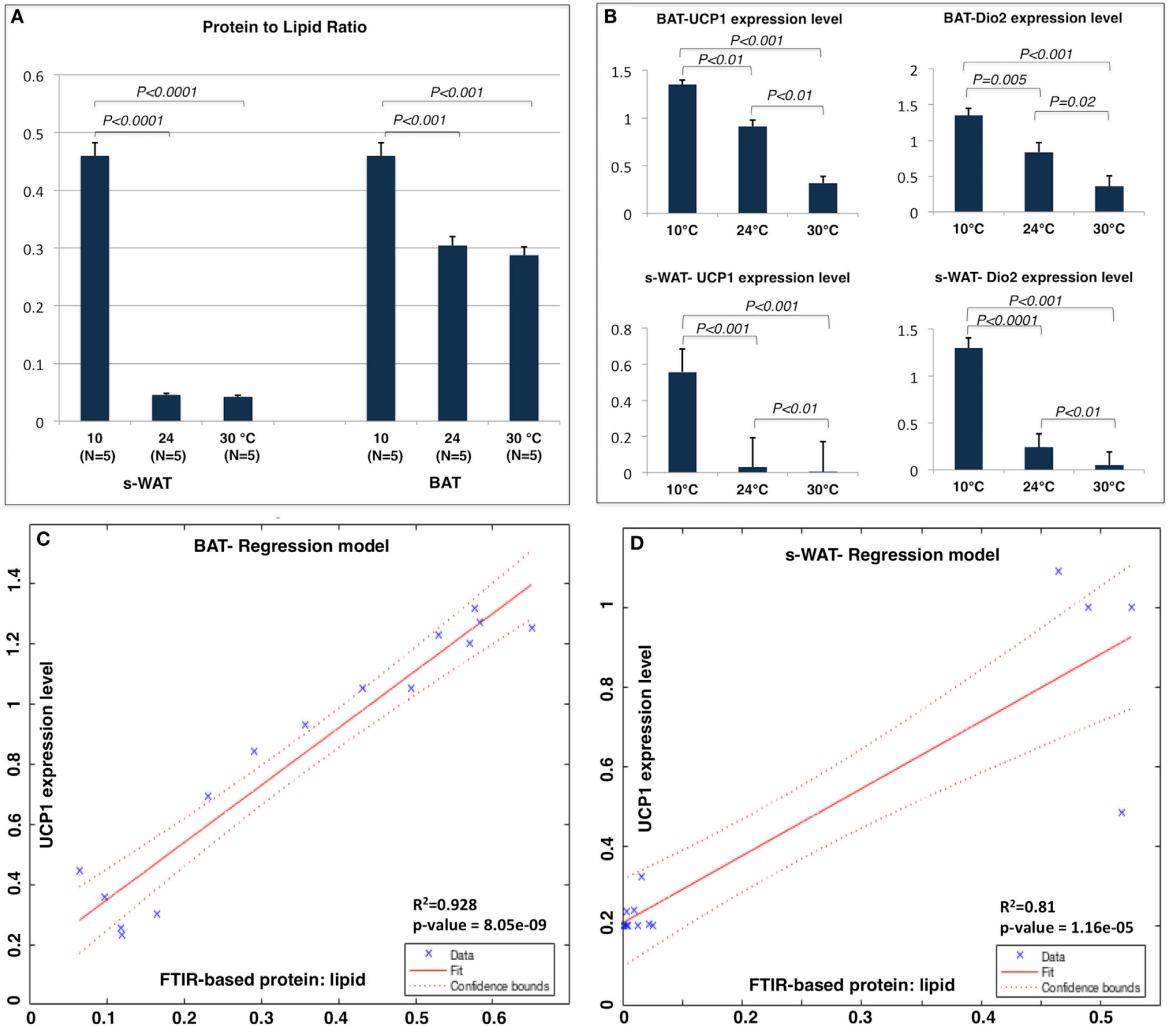
**(A)** Protein to lipid ratio from Fourier transform infrared (FTIR) spectra generated from 10, 24, and 30°C brown adipose tissue (BAT) and subcutaneous white adipose tissue (s-WAT). FTIR spectra were derived from the tiles that were selected for FTIR imaging. Ratio was calculated by dividing the integral of protein band area (1,700–1,600 cm^−1^) to the integral of saturated lipid band area (2,980–2,830 cm^−1^). Stepwise increases in protein to lipid ratio going from 30 to 24 to 10°C BAT (*p* < 0.001) and s-WAT (*p* < 0.0001) were observed. **(B)** The uncoupling protein 1 (UCP-1) and Dio2 expression levels shown in Figure 1 are reshown here for reference to make direct comparison with protein to lipid ratio. Statistical analysis of UCP-1 and Dio2 relative expression levels in BAT and s-WAT at 10, 24, and 30°C are shown. Stepwise increase in UCP-1 level going from 30 to 24 to 10°C BAT (*p* < 0.01) was reported. UCP-1 level in 10°C s-WAT was greater than 24 and 30°C s-WAT (*p* < 0.001). Dio2 level was greater in 10°C BAT and s-WAT than 24°C (*p* = 0.005 and *p* < 0.0001) and 30°C (*p* < 0.001 and *p* < 0.001) BAT and s-WAT. *N* = 5; *N* represents the number of animals per adipose tissue/temperature. **(C,D)** Correlation from linear regression model, suggesting that protein to lipid ratio from FTIR measurements was positively correlated with UCP-1 expression level in BAT (Panel C—*R*^2^ = 92.8%; *p* < 0.0001) and s-WAT (Panel D—*R*^2^ = 81.0%; *p* < 0.0001). The confidence bounds are 95%. Quantitative measurements were derived from multiple analyses of several tissue slices from different animals (*N* = 5) and then combined to provide a value. *p* < 0.05 were accepted as the level of significance and error bars represent 95% confidence intervals.

Figure 4A shows the protein to lipid ratio for 30, 24, and 10°C BAT and s-WAT for all five animals. BAT and s-WAT showed stepwise increases in protein to lipid ratio, going from 30 to 24 to 10°C groups (*p* < 0.01). Protein to lipid ratio was significantly higher in cold-exposed BAT and s-WAT compared to 24, and 30°C tissues (*p* < 0.01). Figure 4B shows quantitative measurements of UCP-1 protein and Dio2 expression level from BAT and s-WAT at 30, 24, and 10°C. There was a stepwise increase going from 30 to 24 to 10°C (*p* < 0.01) in BAT and s-WAT; however, the amount of UCP-1 is negligibly small in 24 and 30°C s-WAT compared to 10°C group, which was consistent with FTIR-based ratios. Quantitative measurements were derived from multiple analyses of several tissue slices from different animals and then averaged to provide a value. In linear regression models, greater values of the protein to lipid ratio were correlated with greater UCP-1 expression level in BAT (*p* < 0.0001, Figure 4C) and s-WAT (*p* < 0.0001, Figure 4D). To generate the relative gene expressions, we divided the absolute value of gene expressions by levels of some cyclophilin that is stable and doesn’t change for the experimental condition and thus serves in essence to control for the amount of RNA in the sample. This is performed for the ease of interpretability, and the results remained unchanged. However, the numbers would have been scaled slightly differently, if we used absolute gene expression values.

**Figure 5:**
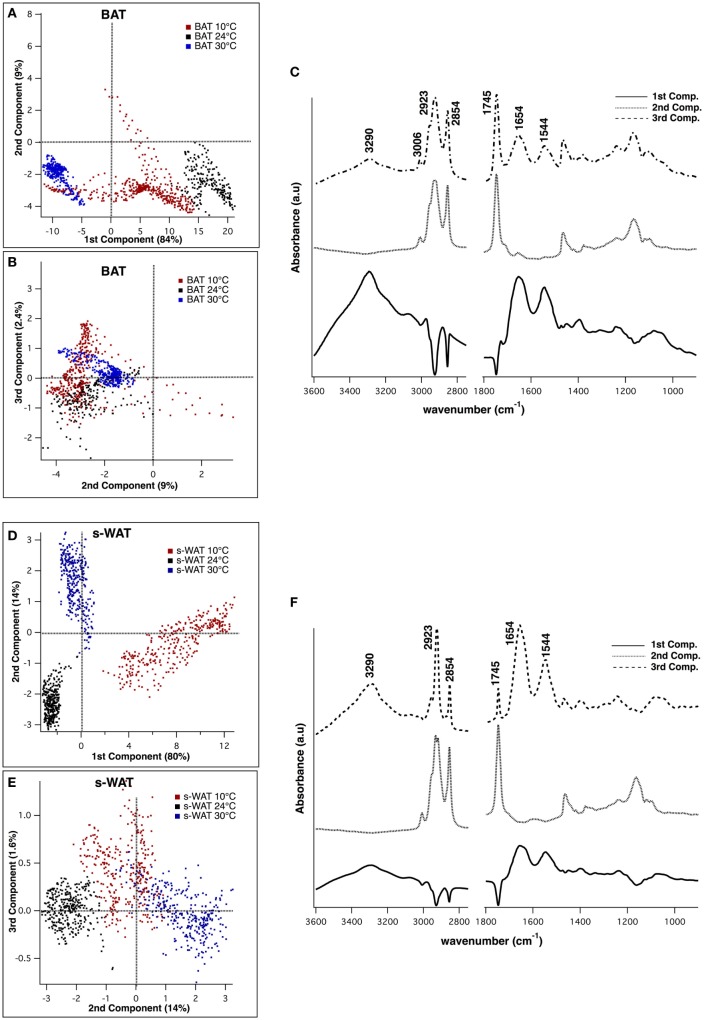
Principal component analysis (PCA) of the spectra derived from Fourier transform infrared (FTIR) imaging data, to demonstrate classification between spectra from 10, 24, and 30°C brown adipose tissue (BAT) **(A–C)** and subcutaneous white adipose tissue (s-WAT) **(D–F)**. Two-dimensional scores scatter plots projected onto the first three principal components, demonstrating classification between the spectra from 10°C (red dots), 24°C (black dots), and 30°C (blue dots) BAT **(A,B)** and s-WAT **(D,E)**. Each dot in the score plot is representative of a pixel (spectrum) within each tile that was selected for imaging. Data are representative of one section per adipose tissue/temperature. 600 spectra from each tissue type/temperature were used to generate the scores plot. Distribution of scores in the scores plot represents the variation of data in that particular group. Loading plot from PCA demonstrates the protein bands (1,544, 1,654, and 3,290 cm^−1^) in the first and the third components, and the lipid bands (3,006, 2,923, 2,854, and 1,745 cm^−1^) in the second component **(C,F)**.

Figure 5 shows the results from PCA applied to 600 individual pixel spectra from BAT and s-WAT at 30, 24, and 10°C. Three PCs that were used to visualize the classification of BAT spectra, represent more than 90% of the variance in the data (Figures 5A,B). Figure 5C shows the first three principal components, where the first component represents three positive bands attributed to proteins (3,290, 1,654, and 1,544 cm^−1^) and three negative bands attributed to C=O ester in phospholipids (1,745 cm^−1^), and symmetric and asymmetric stretching of CH_2_. The second component was only attributed to lipid signatures (3,006, 2,923, 2,854, and 1,745 cm^−1^). The strongest bands in the third component were a combination of protein and lipid bands, very similar to an average spectrum. Figures 5D,E show the projection of data into the first three components for s-WAT, where the scores were well separated between the temperatures. As shown (Figure 5F), the first component shows the strongest positive peaks in protein region (3,290, 1,654, and 1,544 cm^−1^) and the strongest negative peaks in lipids (2,923 and 2,854 cm^−1^) and C=O ester in phospholipids (1,745 cm^−1^). The second and the third principal components were similar to those observed for the analysis of BAT. Overall, we observe a robust distinction between spectra for 30, 24, and 10°C temperatures, within both BAT and s-WAT.

### Unsaturation Level Determined by ^1^H NMR and FTIR Measurements

**Figure 6:**
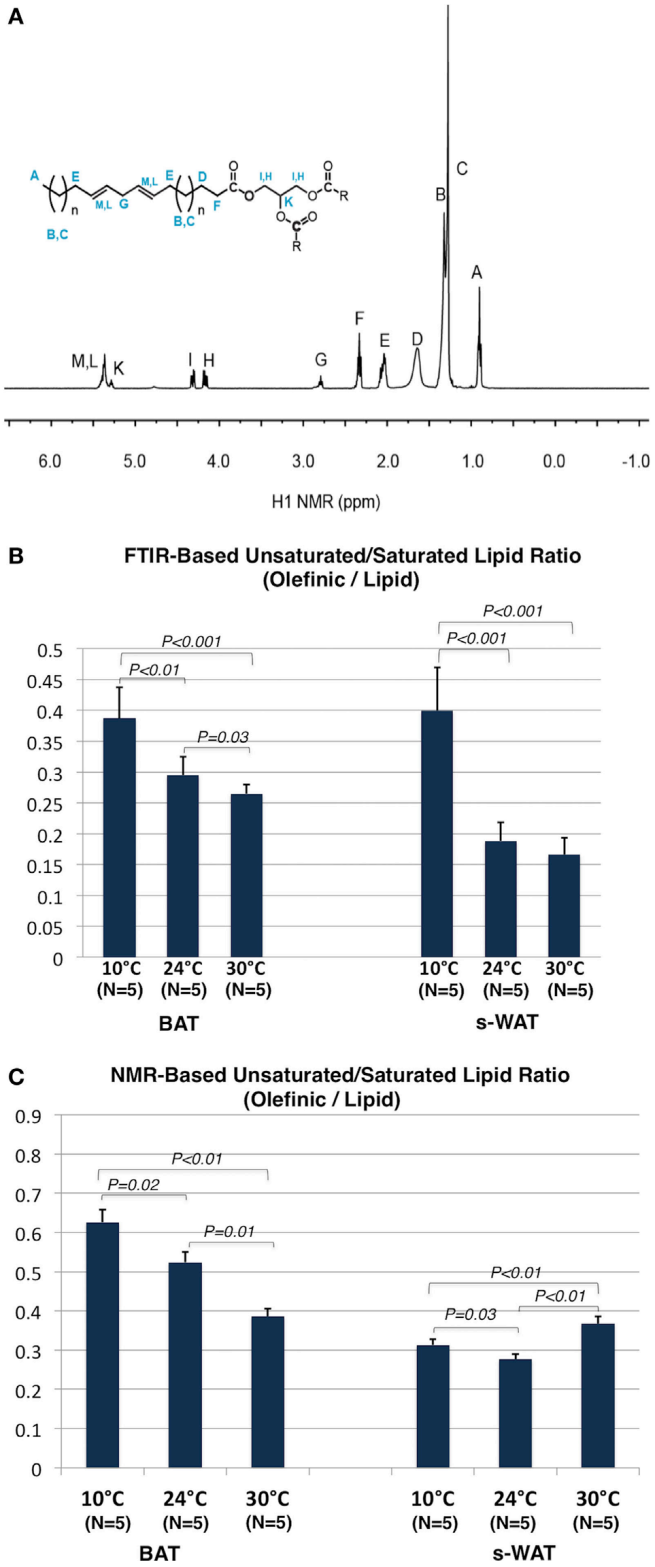
**(A)** ^1^H NMR spectrum of an adipose tissue and the structure of triacylglycerol (TAG). **(B)** Olefinic to lipid ratio from Fourier transform infrared (FTIR) spectra generated from 10, 24, and 30°C tissues, was calculated by dividing the integral of the olefinic band area (3,020–2,992 cm^−1^) to the integral of saturated lipid band area (2,980–2,830 cm^−1^). The same baseline region (3,050–2,750) was used. Stepwise increases in this ratio going from 30 to 24°C (*p* = 0.03) to 10°C (*p* < 0.01) brown adipose tissue (BAT) and subcutaneous white adipose tissue (s-WAT) (*p* < 0.001) were observed. **(C)** Olefinic to lipid ratio from ^1^H NMR spectra generated from 10°C, 24°C, and 30°C BAT and s-WAT, was calculated by dividing the integral of the peak “G” area to the integral of the peak “A” area. Stepwise increase in this ratio from NMR measurements, from 30 to 24°C (*p* = 0.01) to 10°C (*p* = 0.02) BAT was reported. Olefinic to lipid ratio in s-WAT decreased from 30 to 24°C (*p* < 0.01) group and then followed by an increase to 10°C (*p* = 0.03) group. *p* < 0.05 were accepted as the level of significance and error bars represent 95% confidence intervals.

Figure 6A shows a ^1^H NMR spectrum of the adipose tissue and the triacylglycerol (TAG) structure. ^1^H NMR spectra from both BAT and s-WAT reveal signals associated with TAG structure including the hydrogen peaks associated with fatty acids. Peaks attributed to CH_2_, CH_3_, and allylic protons of the fatty acids were observed in the region of 0.87–2.78 ppm (peaks A–E). In the range of 4.13–5.28 ppm, two multiplets assigned to the CH_2_ and CH protons of the glycerol moiety (peaks I and H) and in the range of 5.33–5.41 ppm, a broad multiplet assigned to the vinylic (=CH) protons of the double bonds of the fatty acid chains (peaks M and L) were seen (41). Due to the stability and uniqueness of the peak A in every type of chain, this peak is used as a reference peak. The ratio between integrated signature bonds can be used a tool to determine the level of unsaturation in each group of samples. Figure 6B shows the olefinic to lipid ratio from FTIR measurements, which showed an increase in a stepwise fashion going from 30 to 24 to 10°C groups (*p* < 0.001). Cold-exposed tissues (10°C) showed a significantly higher level of olefinic to lipid ratio in BAT (*p* < 0.01) and s-WAT (*p* < 0.001). The area under the spectral range 3,020–2,992 cm^−1^ attributed to PUFA to that of the spectral range 2,980–2,830 cm^−1^ associated with saturated fatty acids (SAFAs) was calculated. The same baseline (3,050–2,800 cm^−1^) was chosen for both PUFA and SAFA peaks. Figure 6C shows the olefinic to lipid ratio from ^1^H spectra, defined as the ratio of PUFA functional group denoted as “G” in the spectrum to that of the peak “A,” which is the H of the ending -CH_3_ functional group. The olefinic to lipid ratio from ^1^H spectra, showed a significant increase in BAT going from 30°C to greater levels of cold exposure (10°C); however, remained at the same level with the negligible reduction from 10 to 30°C for s-WAT.

## Discussion

Cold exposure has the potential to increase energy expenditure within adipose tissue during weight maintenance and thereby attenuate metabolic adaptation. To demonstrate the ability of these agents to activate adipose tissues, a suitable imaging approach is needed to quantify the corresponding alterations in the biochemical properties of adipose tissues. In this article, we demonstrate how non-destructive, label-free FTIR imaging is able to capture biomolecular and structural alterations within BAT and s-WAT caused by one fat activating manipulation (cold exposure) that is known to increase energy expenditure within these tissues. FTIR imaging identified differences in protein and lipid content in cold-exposed tissues that correlates well with mRNA markers of fat activation and differs by degree of cold exposure. That these mRNA markers are modulated by cold exposure is well established through several prior studies (2, 42–46).

Although histological staining is a common approach to studying the composition of adipose tissues, the application of fixatives and stains could confound the measurement of carbohydrate, protein, and lipid content (47). Instead, FTIR uses inherent spectroscopic properties of biochemical constituents and molecular vibrations to provide information about the concentration and localization of these important macromolecules that is complementary to information provided by fixation and staining (Figure 3). The ability to visualize the spatial distribution of these macromolecules proved especially important in adipose tissue, which shows marked spatial heterogeneities in protein content (Figure 3—Panel B). Therefore, FTIR could provide unique value to molecular examinations of adipose tissue, in combination with classical fixation and staining techniques.

We observed that the protein to lipid ratio is greater in mice exposed to greater degrees of cold, and we identified correlations between greater protein to lipid ratio and greater expression of mRNA markers of fat activation. The ratio of signals from protein and lipid absorption bands gives information about the relative protein content in the tissue. There are several possible biological sources for increased protein content in cold-exposed adipose tissue. Cold exposure can lead to increased mitochondrial content within adipocytes, proliferation of non-adipocyte constituents of the adipose tissue (including fibroblasts, macrophages, and vascular endothelial cells), and increased sympathetic innervation and angiogenesis (48–50). Each of these changes could increase the protein content of the adipose tissue (51, 52). Future research should examine how each of these adipose tissue constituents contributes to the marked differences in relative protein content seen in cold exposure.

We used two complementary methods, FTIR and ^1^H NMR, to calculate the olefinic to lipid ratio, a proxy measure of the relative concentration of unsaturated, as opposed to saturated, lipids in the adipose tissues. In BAT, both modalities showed stepwise increases with stepwise increases in the degree of cold exposure. FTIR suggested that 10°C WAT had greater unsaturated lipid content than the warmer temperatures, while NMR suggested only minor differences in unsaturated lipid content by temperature. The agreement between FTIR and ^1^H NMR in BAT provides validation of the FTIR methodology in this tissue. In addition, unsaturated lipids play a major role in cellular and physiological processes such as cold adaptation (53), suggesting they should be present in greater abundance in cold exposed tissues. The difference between FTIR and NMR in s-WAT could be due to the fact that NMR provides data at the level of bulk tissues while FTIR provides spatially localized information. Spatial heterogeneity in unsaturated lipid content in colder tissues may have been captured well by our localized FTIR analysis, and averaged out of the NMR measurements.

In conclusion, we have shown the ability of FTIR imaging to quantify biochemical changes due to cold exposure in regions of functionally active BAT and s-WAT of mice. FTIR was able to quantify protein and lipid content in a label-free manner that is complementary to staining and fixation techniques. Because it is a non-destructive, label-free technique, future work could extend FTIR to longitudinal *in vivo* measurements over an extended time period. For example, FTIR could be a viable technology for ecological, momentary assessment of fat activation level through mid-IR wearable sensors. Such sensors could enable FTIR to shed important insight into the potential role of fat activation as a therapeutic target for the treatment of obesity and diabetes.

## Author Contributions

EA performed Cryosectioning, FTIR data acquisition, multivariate analysis, and data interpretation. PH analyzed and contributed ^1^H NMR data. DA contributed animal preparations and real-time PCR measurements. OC, CM, and CH designed, supervised, and discussed the results and implications of the research. OC and CH contributed to the final approval of the research and interpretation of data. OC and CH contributed equally as senior authors. All authors made contributions writing the article.

## Conflict of Interest Statement

The authors declare that the research was conducted in the absence of any commercial or financial relationships that could be construed as a potential conflict of interest.
